# Anti-Biofilm Properties of *Saccharomyces cerevisiae* CNCM I-3856 and *Lacticaseibacillus rhamnosus* ATCC 53103 Probiotics against *G. vaginalis*

**DOI:** 10.3390/microorganisms8091294

**Published:** 2020-08-24

**Authors:** Samuele Sabbatini, Claudia Monari, Nathalie Ballet, Amélie Cayzeele Decherf, Silvia Bozza, Barbara Camilloni, Stefano Perito, Anna Vecchiarelli

**Affiliations:** 1 Department of Medicine, Medical Microbiology Section, University of Perugia, Polo Unico Sant’Andrea delle Fratte, 06132, Perugia, Italy; samuele.sabbatini@gmail.com (S.S.); silvia.bozza@unipg.it (S.B.); barbara.camilloni@unipg.it (B.C.); stefano.perito@unipg.it (S.P.); anna.vecchiarelli@unipg.it (A.V.); 2 Lesaffre International, Lesaffre Group, Rue Gabriel Péri 137, Marcq-en-Baroeul, 59700, France; n.ballet@lesaffre.com; 3 Gnosis by Lesaffre, Lesaffre Group, Rue Gabriel Péri 137, Marcq-en-Baroeul, 59700, France; a.decherf@gnosis.lesaffre.com

**Keywords:** probiotics, *Saccharomyces cerevisiae*, biofilm, *Gardnerella vaginalis*, bacterial vaginosis, *Lacticaseibacillus rhamnosus*, metronidazole

## Abstract

Bacterial vaginosis (BV) is characterized by the presence of a polymicrobial biofilm where *Gardnerella vaginalis* plays a key role. Previously, we demonstrated that *Saccharomyces cerevisiae* CNCM (French National Collection of Cultures of Microorganisms) I-3856 is helpful in resolving experimental simulated BV in mice. In this study, we analyzed its capacity to affect *G. vaginalis* biofilms and to potentiate the activity of standard antimicrobial agents. We also investigated the anti-biofilm activity of *Lacticaseibacillus rhamnosus* GG (ATCC 53103), a well-known strain for its intestinal healthy benefits. Biofilm biomass was assessed by crystal violet staining, and *G. vaginalis* viability was assessed by a colony forming unit (CFU) assay. Here, for the first time, we demonstrated that *S. cerevisiae* CNCM I-3856 as well as *L. rhamnosus* GG were able (i) to significantly inhibit *G. vaginalis* biofilm formation, (ii) to markedly reduce *G. vaginalis* viability among the biomass constituting the biofilm, (iii) to induce disaggregation of preformed biofilm, and (iv) to kill a consistent amount of bacterial cells in a *G. vaginalis* preformed biofilm. Furthermore, *S. cerevisiae* CNCM I-3856 strongly potentiates the metronidazole effect on *G. vaginalis* biofilm viability. These results suggest that *S. cerevisiae* CNCM I-3856 as well as *L. rhamnosus* GG could be potential novel therapeutic agents against bacterial vaginosis.

## 1. Introduction

Bacterial vaginosis (BV) is the most common vaginal dysbiosis affecting fertile, pregnant, and premenopausal women [1]. BV has been associated with serious obstetrical and gynecological complications such as spontaneous abortion [2], preterm birth [3], endometritis [4], pelvic inflammatory disease [5], postoperative infections [6], and acquisition of sexually transmitted infections [7] such as human immunodeficiency virus (HIV) [8].

The clinical symptoms of vaginosis include profuse vaginal discharge, itching, burning, and a rotten fish vaginal odor. Nevertheless, many women with BV remain asymptomatic. Among a representative sample of women of reproductive age in the United States, Koumas and colleagues determined that 84% of women with BV did not report symptoms [9]. Bacterial vaginosis is typically associated with a dramatic reduction of healthy vaginal microbiota, constituted mainly by beneficial D-lactic acid and hydrogen peroxide-producing lactobacilli such as *Lactobacillus crispatus*, that plays an important role in host defense against pathogens [10,11], and by a simultaneous overgrowth of anaerobic pathogenic bacteria including *Gardnerella vaginalis* (*G. vaginalis*), *Prevotella* spp.,* Atopobium vaginae (A. vaginae)*, *Bacteroides* spp., and *Mobiluncus* spp. [12,13]. In addition, recently, it has been reported that not all *Lactobacillus* spp. decrease during BV. In vaginal swabs from premenopausal Caucasian women with bacterial vaginosis, *L. crispatus* was progressively replaced by *L. iners* [11] that, being unable to produce hydrogen peroxide and D-lactic acid, seems to have a lower protective capacity with respect to the other *Lactobacillus* spp. [14].

To date, the consensus is that a polymicrobial structured biofilm is usually present on the vaginal epithelium of women with BV. Current data suggest that *G. vaginalis* plays a key role in BV pathogenesis, showing higher initial adhesion, damage, and apoptosis of vaginal epithelial cells and a greater biofilm-producing capacity compared to the other BV-associated anaerobes [15,16,17,18].

The biofilm structure prevents the possibility of antimicrobials agents reaching the site of infection [19]. This latter activity is one of the most important characteristics of a biofilm [20,21]. It has been demonstrated that microorganisms composing a biofilm can be up to 1000 times more resistant to most antibiotics as compared to when they are in their planktonic growth phase [22].

To date, conventional therapeutic strategies available for BV include treatments with metronidazole, clindamycin, or tinidazole. Metronidazole is considered the drug of choice [23,24]. However, these standard antibiotic therapies seem to be unable to fully eradicate BV vaginal biofilms, leading to high rates of BV recurrences. A recurrence rate of BV up to 60% within 12 months from the onset of therapeutic treatment was observed [25,26]. Possible reasons for the failure of conventional treatments and for high recurrence rates of BV could be the strong polymicrobial biofilm present in 90% of women with BV [27,28] as well as the *G. vaginalis* internalization by vaginal epithelial cells that may allow *G. vaginalis* to escape from the antibiotic effect [29]. 

Moreover, antibiotic treatments and especially the prolonged and repeated ones support the development of resistant pathogens [18,23,27]. Alves and colleagues determined the in vitro susceptibility of 30 BV-associated species to metronidazole, tinidazole, and clindamycin and showed that all tested strains were resistant to metronidazole and tinidazole whereas 20 strains were resistant to clindamycin [30]. The need for alternative compounds that could be used either alone or in combination with antibiotics to prevent and/or to treat BV has led to the development of new therapeutic approaches both to inhibit the formation and/or to degrade an established biofilm [18]. Several compounds, including antibacterial enzymes and peptides, antiseptics and tensides [26,31], cationic amphiphiles [32], DNAse-targeting extracellular polymeric substance [33], and quorum-sensing inhibitors subtilosin [32] and benzoyl peroxide [34] have been tested. To date, although some of them look promising, clinical evaluation is quite limited. Recently, Gottschick et al. [35], in a controlled randomized clinical trial, demonstrated that an amphoteric tenside agent (WO3191) was able to reduce biofilm but not to prevent recurrence. Other promising adjuvant or alternative approaches involve the administration of probiotics, i.e., live microorganisms [36]. At present, although in recent years probiotic-based strategies have been shown to be a promising tool for both prophylaxis and treatment of BV [37,38,39,40], their role in preventing biofilm formation and/or in dispersing *G. vaginalis* biofilms has been poorly investigated.

*Saccharomyces cerevisiae* (*S. cerevisiae*) and the lactic acid bacterium *Lacticaseibacillus rhamnosus* (*L. rhamnosus*) are among the most studied probiotics [39,40,41,42,43,44,45,46,47,48]. Recently, we demonstrated, for the first time, that the probiotic yeast *S. cerevisiae* CNCM (French National Collection of Cultures of Microorganisms) I-3856 shows a beneficial effect in resolving experimental BV in mice [49] by interference with *G. vaginalis* adherence to vaginal epithelial cells (ECs), displacement of *G. vaginalis* attached to ECs, inhibition of sialidase activity, and reduction of vaginal epithelial exfoliation. *L. rhamnosus* GG is one of the probiotic strains with the largest number of documented health benefits, mainly regarding prevention and treatment of intestinal tract infections [50]. Recently, it was demonstrated that *L. rhamnosus* GG was able to inhibit *C. albicans* and *C. glabrata* adhesion to the vaginal epithelial cell line VK2/E6E7 in both competition and displacement assays [39]. Many *L. rhamnosus* strains have been shown to exert a protective effect against bacterial vaginosis [39]. To our knowledge, the capacity of *L. rhamnosus* GG to counteract this infection has not been investigated.

The aim of the current study was to evaluate the ability of *S. cerevisiae* CNCM I-3856 (GI) and of *L. rhamnosus* GG (G 250) to inhibit *G. vaginalis* biofilm formation and/or to disaggregate preformed *G. vaginalis* biofilm. We also tested if *S. cerevisiae* CNCM I-3856 was able to potentiate the effects of metronidazole or clindamycin on mature *G. vaginalis* biofilm.

## 2. Materials and Methods

### 2.1. Study Products

The compounds studied were provided by Gnosis by Lesaffre, a Business Unit of the Lesaffre Group (Marcq-en-Baroeul, France). *S. cerevisiae* live yeast (referenced GI) is a proprietary, well-characterized strain of Lesaffre registered in the French National Collection of Cultures of Microorganisms (CNCM) under the number I-3856. The *S. cerevisiae* species was determined by using phenotypic (API^®^ID32C, Biomerieux Marcy-l’Étoile, France) and genotypic referenced methods (genetic amplification and sequencing of 26S DNA) [51,52]. Moreover, the strain CNCM I-3856 has been characterized by polymerase chain reaction (PCR) Interdelta typing techniques [53] and other genetic methods (e.g., complete genome sequencing). Furthermore, the strain of *L. rhamnosus* GG (referenced as G 250) is registered in the American Type Culture Collection (ATCC) under the number 53103, and it can be taken as a dietary supplement [54]. Since April 2020, *Lactobacillus rhamnosus* has been officially reclassified to *Lacticaseibacillus rhamnosus (L. rhamnosus)* [55].

### 2.2. Microbial Strains and Growth Conditions

*G. vaginalis* clinical isolate was obtained from a vaginal swab from the Microbiology Unit of Santa Maria della Misericordia Hospital of Perugia (Italy). The swab was immediately used to inoculate Gardnerella selective agar (GSA) media (plates with 5% of human blood, Becton and Dickinson, Franklin Lakes, NJ, USA). The plates were incubated anaerobically at 37 °C for 24–48 h, ß-hemolytic colonies were isolated, and candidate *G. vaginalis* strains were identified by Matrix-Assisted Laser Desorption/Ionization Time-of-Flight (MALDI-TOF, Bruker Daltonics, Billerica, MA, USA) mass spectrometry [17,49].

A spontaneous streptomycin-resistant mutant was isolated by plating *G. vaginalis* on New York City III (NYC-III) agar plates +1 mg/mL streptomycin (Sigma-Aldrich) and by selecting resistant colonies after incubating anaerobically at 37 °C for 72 h [49]. Streptomycin-resistant *G. vaginalis* was cultured in supplemented Brain Hearth Infusion broth (sBHI, Oxoid, St. Louis, MO, USA) with 2% (wt/v) gelatin (Sigma-Aldrich, St. Louis, MO, USA), 1% (wt/v) yeast extract (Sigma-Aldrich, St. Louis, MO, USA), 0.1% (wt/v) soluble starch (Sigma-Aldrich, St. Louis, MO, USA), and 0.25% (wt/v) maltose (Panreac, Barcelona, Spain) and was incubated anaerobically at 37 °C. Glycerol stock cultures of *G. vaginalis* were stored in sBHI at −80 °C. The resistant *G. vaginalis* mutant has been used for all experiments.

Before each experiment, *G. vaginalis* was harvested by centrifugation for 5 min at 11,000 rpm and washed twice with sterile phosphate-buffered saline (PBS, Life Technologies, Carlsbad, CA, USA), and the concentration was adjusted to that desired and suspended in the appropriate buffer.

### 2.3. Biofilm Cultures

A pre-culture was started from the *G. vaginalis* glycerol stock in sBHI and incubated overnight at 37 °C with 10% CO_2_. Then, bacterial density was adjusted to 10^8^ colony forming units (CFU)/mL, and 100 µL was transferred to each well of 96-well plates. Plates were then incubated at 37 °C with 10% CO_2_ for 14, 18, 24, or 48 h [56]. To obtain a mature and stable biofilm [56], the culture medium was replaced by fresh medium after 24 h of growth and the biofilm was incubated for another 24 h. For probiotic biofilm formation, different concentrations of GI or G 250 (10^9^–10^7^ CFU/mL) were incubated in sBHI for 24 h at 37 °C with 10% CO_2_.

### 2.4. Inhibition and Disaggregation of G. vaginalis Biofilm 

To perform biofilm formation inhibition experiments, an overnight culture of *G. vaginalis* in sBHI was adjusted to 10^8^ CFU/mL, 100 µL of this suspension was transferred to each well of a 96-well microplate, and 100 µL of GI or G 250 (10^9^–10^7^ CFU/mL) was added at the beginning of the biofilm culture. The analyses were carried out after 24 h of incubation at 37 °C with 10% CO_2_. For the biofilm disaggregation assay, an overnight culture of *G. vaginalis* in sBHI was adjusted to 10^8^ CFU/mL, 100 µL of this suspension was transferred to each well, and the plate was incubated at 37 °C with 10% CO_2_ for 24 h. After incubation, the medium was removed and a fresh sBHI medium containing different concentrations of probiotics GI or G 250 (10^9^–10^7^ CFU/mL) was added to the preformed biofilm and incubated for another 24 h under the same conditions as above described. In the selected experiments, GI in combination with metronidazole or clindamycin were added to the preformed biofilm and *G. vaginalis* viability was evaluated. Biofilm biomass was determined by the crystal violet (CV) staining method, and *G. vaginalis* biofilm viability was determined by a CFU assay.

### 2.5. Quantification of Biofilm Biomass and Biofilm Viability

Biofilm biomass was quantified using the CV staining method [56]. After the appropriate incubation time, the biofilm was washed twice with 200 µL of PBS and fixed with 100 µl of 99% methanol. After 15 min, supernatants were removed, the plates were air dried, and then biofilms were stained with 100 µL of 0.5% (wt/v) CV (Sigma-Aldrich, St. Louis, MO, USA) for 20 min. Afterwards, the plates were washed twice with 200 µL of PBS to remove excess CV. Finally, CV was solubilized by adding 150 µL of 33% (v/v) acetic acid per well. The optical density (OD) at 590 nm was measured using a 96-well microplate reader (Tecan, Männedorf, Switzerland). 

Biofilm viability was determined by CFU determination [26]. After the appropriate incubation time, biofilm was washed twice with 200 µl of PBS, scraped off, and suspended in 50 µL of 0.85% NaCl before serial dilution and spreading onto NYC III agar plates with 1 mg/mL streptomycin and 4 mg/mL amphotericin B (Sigma-Aldrich, St. Louis, MO, USA). The plates were incubated at 37 °C with 10% CO_2_ for 48 h prior to CFU count. *G. vaginalis* biofilm viability as consequence of coincubation with GI or G 250 was calculated as a percentage of *G. vaginalis* CFU from dual-species biofilm with respect to those from *G. vaginalis* monomicrobial biofilms.

The percentage of *G. vaginalis* biofilm viability reduction as a consequence of treatment with GI or G 250 was calculated as follows: 100 – (100 × (*G. vaginalis*-treated group/*G. vaginalis* untreated group)) [26,57].

### 2.6. Co-Aggregation Assay

The co-aggregation assay was performed as previously described [2,49]. Briefly, *G. vaginalis* (10^9^ CFU/mL) or fluorescein isothiocyanate-labeled *G. vaginalis* (FITC-*G. vaginalis*, 10^9^ CFU/mL) in PBS were mixed with equal volumes of G 250 (10^9^ CFU/mL) or Rhodamine B-labeled G 250 (RHB-G 250, 10^9^ CFU/mL). Then, the samples were vortexed for at least 10 sec and incubated in 24-well plates for 4 h at 37 °C under agitation. The suspensions were then observed by inversion light microscopy to evaluate the aggregation degree and scored according to the following scale: 0 = no aggregation; 1 = small aggregates comprising small visible clusters; 2 = aggregates comprising larger numbers of microorganisms, settling down to the center of the well; 3 = macroscopically visible clumps comprising larger groups which settle to the center of the well; and 4 = maximum score allocated to describe a large, macroscopically visible clump in the center of the well [49]. Moreover, each fluorescent suspension was analyzed under a fluorescent light microscope (Carl Zeiss) [58].

### 2.7. Anti-Biofilm Activity of Metronidazole and Clindamycin

The antibiotics metronidazole and clindamycin were purchased from Sigma-Aldrich. Metronidazole stock solution was dissolved in 0.1 M acetic acid solution, filter-sterilized, and directly diluted before each experiment. Stock solutions of clindamycin (1 mg/mL) were dissolved in sterile H_2_O, filter-sterilized, and kept at −20 °C.

The minimal inhibitory concentration (MIC) of antibiotics metronidazole and clindamycin was assessed using the broth microdilution method according to Sutyak Noll et al. [59] with some modifications. Stock solutions of the antimicrobials were serially diluted with grown medium to the appropriate concentrations, and 100 µL of each dilution was added to a 96-well plate (Corning). *G. vaginalis* was cultured overnight and diluted with fresh medium to a concentration of 5 × 10^6^ CFU/mL, and 100 µL was transferred to the wells containing the antibiotics. *G. vaginalis* alone and growth medium alone were used as controls. The plate was incubated under anaerobic conditions at 37 °C for 24–48 h. The MIC was defined as the lowest concentration of the antibiotics that showed no bacterial growth. To determine the minimal biofilm bactericidal concentration (BBC), defined as the lowest concentration that killed 99.9% of the cells recovered from a biofilm culture compared to growth control [21], the medium of a 24 h biofilm of *G. vaginalis* was removed, fresh sBHI medium containing two-fold serial dilutions of metronidazole (concentrations range: 64–4 µg/mL) and clindamycin (concentrations range: 0.5–0.0312 µg/mL) was added, and the biofilm was incubated at 37 °C with 10% CO_2_ for 24 h. After the incubation, biofilm viability was determined by a CFU assay [26].

### 2.8. Statistical Analysis

The results reported are the means ± standard error of the mean (SEM) of triplicate samples from 3 to 5 different experiments. Data were evaluated using ANOVA. Post hoc comparisons were done with Bonferroni’s test. A value of *p* < 0.05 was considered significant.

## 3. Results

### 3.1. Effect of S. cerevisiae CNCM I-3856 (GI) and L. rhamnosus GG (G 250) on G. vaginalis Biofilm Formation

**Figure 1:**
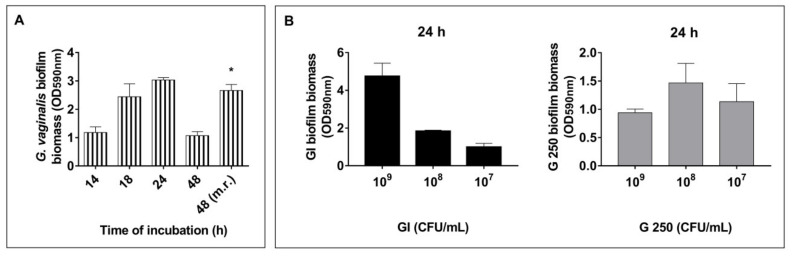
*Gardnerella vaginalis*, *Saccharomyces cerevisiae* CNCM (French National Collection of Cultures of Microorganisms) I-3856 (GI), and *Lacticaseibacillus rhamnosus* GG (G 250) biofilm formation: (**A**) *G. vaginalis* (10^8^ colony forming units (CFU)/mL) biofilm formation after 14, 18, 24, and 48 h of incubation. For the 48 h m.r. (medium renewal) value, the medium was replaced by a fresh medium after 24 h of incubation. (**B**) Biofilm formation by GI and G 250 (10^9^, 10^8^, and 10^7^ CFU/mL) after 24 h. The biofilm biomass was measured by crystal violet staining (OD, optical density). Results are the mean ± SEM from 3 independent experiments (each with n = 3). * *p* < 0.05 48 h (m.r.) *G. vaginalis* biofilm vs. 48 h *G. vaginalis* biofilm. Data are the mean ± SEM from 4 independent experiments (each with n = 3).

Firstly, the ability of a clinical isolate of *G. vaginalis* to develop a biofilm in our experimental conditions was evaluated. To this end, *G. vaginalis* (10^8^ CFU/mL) was incubated for 14, 18, 24, and 48 h as described in the Materials and Methods section. Biofilm formation on an abiotic surface (polystyrene plates) was determined by CV biomass staining. The results, reported in Figure 1A, show that biofilm formation was observed up till 18–24 h of incubation and that the prolongation of incubation time resulted in a strong decrease of biofilm biomass, showing that *G. vaginalis* biofilm disintegration occurs from 24 h. This decrease was prevented by replacing the spent medium with fresh medium after 24 h (Figure 1A), as shown by the OD value obtained at 48 h medium renewal (m.r.).

It has been reported that biofilm formation by probiotic bacteria is considered a beneficial property because it could promote colonization and longer persistence on the mucosa of the host, thus inhibiting the growth of pathogenic bacteria [60]. As *Saccharomyces* spp. and *Lactobacillus* spp. have previously been shown to be able to form biofilm on abiotic surfaces [60,61], we tested the ability of GI and G 250 to form biofilm in our experimental system. The two probiotics were incubated for 24 h, as described in the Material and Methods section, and the CV staining method was used for biofilm biomass quantification. The results show that both probiotics were able to form biofilm on abiotic surfaces at all concentrations tested. In particular, GI produces biofilm in a dose-dependent manner whereas G 250 did not (Figure 1B).

**Figure 2:**
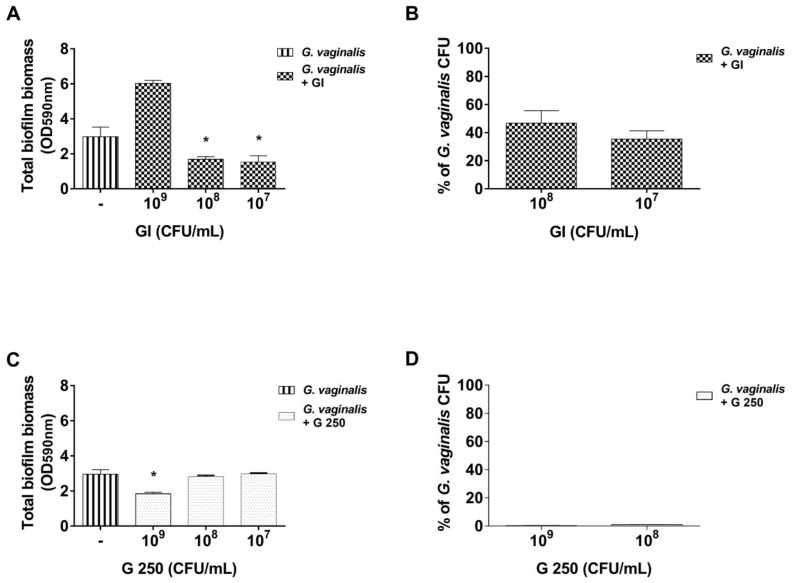
Effect of *S. cerevisiae* CNCM I-3856 (GI) and *L. rhamnosus* GG (G 250) on *G. vaginalis* biofilm formation: *G. vaginalis* (10^8^ CFU/mL) was incubated in presence or absence of GI (**A**,**B**) or G 250 (**C**,**D**), both at 10^9^–10^7^ CFU/mL for 24 h as described in the Materials and Methods section. Then, the following were determined: (**A**,**C**) biofilm biomass development and (**B**,**D**) percentage of *G. vaginalis* CFU recovered from dual-species biofilm with respect to the CFU from a *G. vaginalis* single-species biofilm. Biofilm formation has been evaluated by crystal violet staining (OD, optical density). Data are the mean ± SEM from 4 independent experiments (each with n = 3) for biomass determination and one experiment (n = 3) for CFU evaluation. * *p* < 0.05 probiotic (GI or G 250)–*G. vaginalis* biofilm vs. *G. vaginalis* monomicrobial biofilm.

The capacity of probiotics to inhibit *G. vaginalis* biofilm formation was then evaluated. To this end, GI, and G 250, at concentrations of 10^9^, 10^8^, and 10^7^ CFU/mL, were mixed with *G. vaginalis* (10^8^ CFU/mL) and biofilm development was evaluated after 24 h of incubation. The results show that, in the presence of GI at doses of 10^7^ and 10^6^ CFU/mL, the total biofilm biomass was significantly decreased compared with that of *G. vaginalis* mono-species biofilm (*p* < 0.05, Figure 2A), whereas at the dose of 10^9^ CFU/mL, GI induced an increase in the overall mass of the biofilm, suggesting that the interaction between *G. vaginalis* and probiotics could have stimulated the growth of *G. vaginalis*, of the probiotic, or of both.

To determine the effect of GI on *G. vaginalis* viability in the dual-species biofilm, a CFU assay was performed by using the two concentrations that produced a significant reduction of total biofilm biomass compared to that of *G. vaginalis* alone. The results reported in Figure 2B show that both 10^8^ and 10^7^ CFU/mL of GI produced a strong reduction of *G. vaginalis* viability.

Regarding the effect of G 250 on *G. vaginalis* biofilm development, the results show that G 250 was able to cause a consistent inhibition of biofilm formation at a dose of 10^8^ CFU/mL whereas no inhibition was observed at doses of 10^8^ and 10^7^ CFU/mL (Figure 2C).

To determine the effect of G 250 on *G. vaginalis* viability, a CFU assay was performed by using two doses: 10^9^ that causes inhibition of total biofilm biomass formation and 10^8^ that, like the dose of 10^7^, did not. Of note, both the concentrations were able to achieve near-complete reduction of *G. vaginalis* viability (Figure 2D).

### 3.2. Co-Aggregation between L. rhamnosus GG (G250) and G. vaginalis

Co-aggregation is an important mechanism that influences development of complex multispecies biofilms [62] and can be involved in pathogens elimination by probiotics [63,64,65]. We recently demonstrated that GI does not induce *G. vaginalis* co-aggregation [49]. In this study, we analyzed if G 250 can co-aggregate with *G. vaginalis*. To this end, G 250 was incubated alone or mixed with *G. vaginalis*, as described in the Materials and Methods section. The results reported in Table 1 show that a low level of self-aggregation was manifested by G 250 while the strongest co-aggregation was observed with *G. vaginalis*.

**Figure 3:**
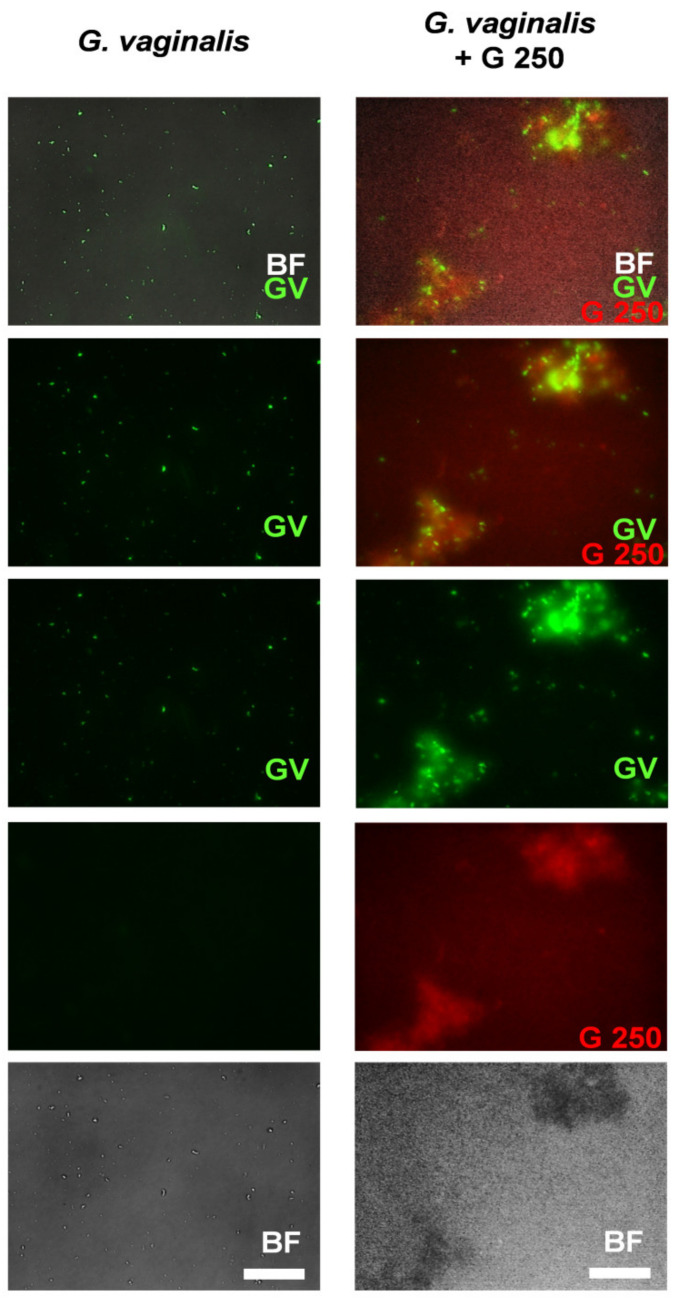
Co-aggregation between *L. rhamnosus* GG (G 250) and *G. vaginalis: G. vaginalis* or fluorescein isothiocyanate-labeled (FITC)-*G. vaginalis* (10^9^ CFU/mL) in 500 μL of phosphate-buffered saline (PBS) were mixed or not with equal volumes of G 250 or RHB-G 250 (G 250) (10^9^ CFU/mL). The samples were vortexed for at least 10 s and incubated in a 24-well plate for 4 h at 37 °C under agitation. The suspensions were then photographed by fluorescence microscopy. Images are from one experiment (scale bar = 50 μm, magnification 20×). BF = bright field; *G. vaginalis* (GV) = green; and G 250 (G 250) = red.

A representative image taken using fluorescence microscopy demonstrating the capacity of probiotic to co-aggregate *G. vaginalis* is shown in Figure 3.

### 3.3. Effect of S. cerevisiae CNCM I-3856 (GI) and L. rhamnosus GG (G 250) on Preformed G. vaginalis Biofilm Disaggregation

**Figure 4:**
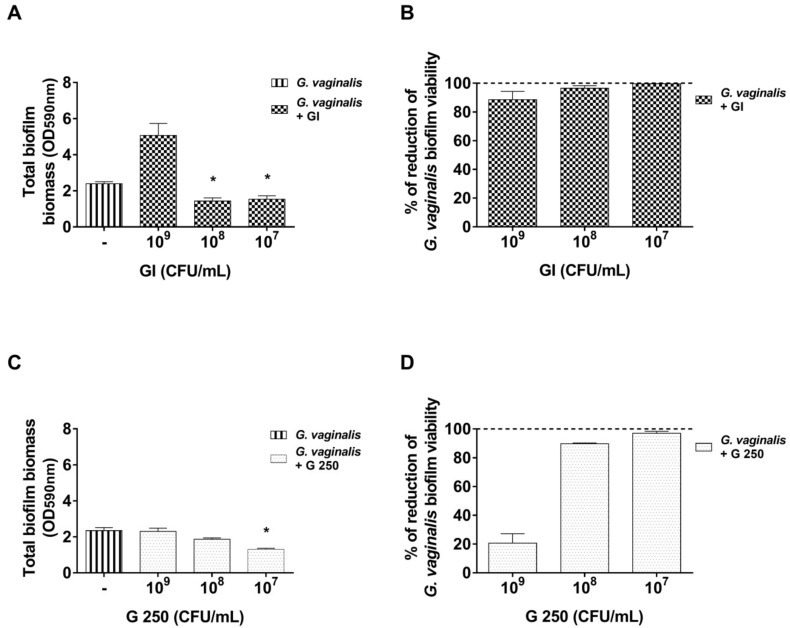
Effect of *S. cerevisiae* CNCM I-3856 (GI) and *L. rhamnosus* GG (G 250) on *G. vaginalis* biofilm disaggregation: *G. vaginalis* (10^8^ CFU/mL) was incubated for 24 h to develop mature biofilm. After incubation, the medium was removed, GI (**A**,**B**) or G 250 (**C**,**D**) were added at different concentrations, and the co-cultures were incubated for a further 24 h as described in the Materials and Methods section. Then, the following were determined: (**A**,**C**) biomass disaggregation and (**B**,**D**) percentage of *G. vaginalis* biofilm viability reduction. Biofilm disaggregation has been evaluated by crystal violet staining (OD, optical density). The *G. vaginalis* biofilm viability has been determined by a colony forming units (CFU) assay. Percentages of biofilm viability reduction probiotics have been quantified with respect to untreated *G. vaginalis* biofilms which were taken as 100% (dotted line). Data are the mean ± SEM from 3 independent experiments (each with n = 3) for biomass determination and from 2 independent experiments (each with n = 2) for viability evaluation. * *p* < 0.05 GI or G 250-treated *G. vaginalis* biofilm vs. untreated *G. vaginalis* biofilm.

To investigate the capacity of probiotics to disaggregate preformed biofilm, *G. vaginalis* (10^8^ CFU/mL) was incubated for 24 h. Then, to avoid physiological disaggregation of a *G. vaginalis* biofilm (see Figure 1A), the medium was removed and fresh sBHI medium alone or containing GI or G 250 (10^9^–10^7^ CFU/mL) was added to the culture. After another 24 h of incubation, the biomass quantification and *G. vaginalis* biofilm viability were determined by the CV staining method and by a CFU assay, respectively. Our results show that GI at the doses of 10^8^ and 10^7^ CFU/mL significantly reduced biofilm biomass compared to the control (*p* < 0.05, Figure 4A).

Of note, all the doses tested (10^9^–10^7^ CFU/mL) killed almost 100% of *G. vaginalis* cells in the remaining biofilm (Figure 4B). Concerning G 250, our data show that it was able to induce a significant disaggregation of the preformed *G. vaginalis* biofilm only at 10^7^ CFU/mL (*p* < 0.05, Figure 4C). The determination of G 250 effect on *G. vaginalis* biofilm viability showed that both 10^8^ and 10^7^ CFU/mL greatly reduced the viability of *G. vaginalis* (Figure 4D).

In subsequent experiments, we also evaluated if GI in association with suboptimal doses of metronidazole or clindamycin could be able to potentiate the antibiotic effect on mature *G. vaginalis* biofilm.

### 3.4. Effect of S. cerevisiae CNCM I-3856 (GI) Association with Metronidazole or Clindamycin on Preformed G. vaginalis Biofilm

To date, standard antibiotic therapies for *G. vaginalis* infection, metronidazole, and clindamycin, seem to be unable to fully eradicate BV vaginal biofilms [23].

**Figure 5:**
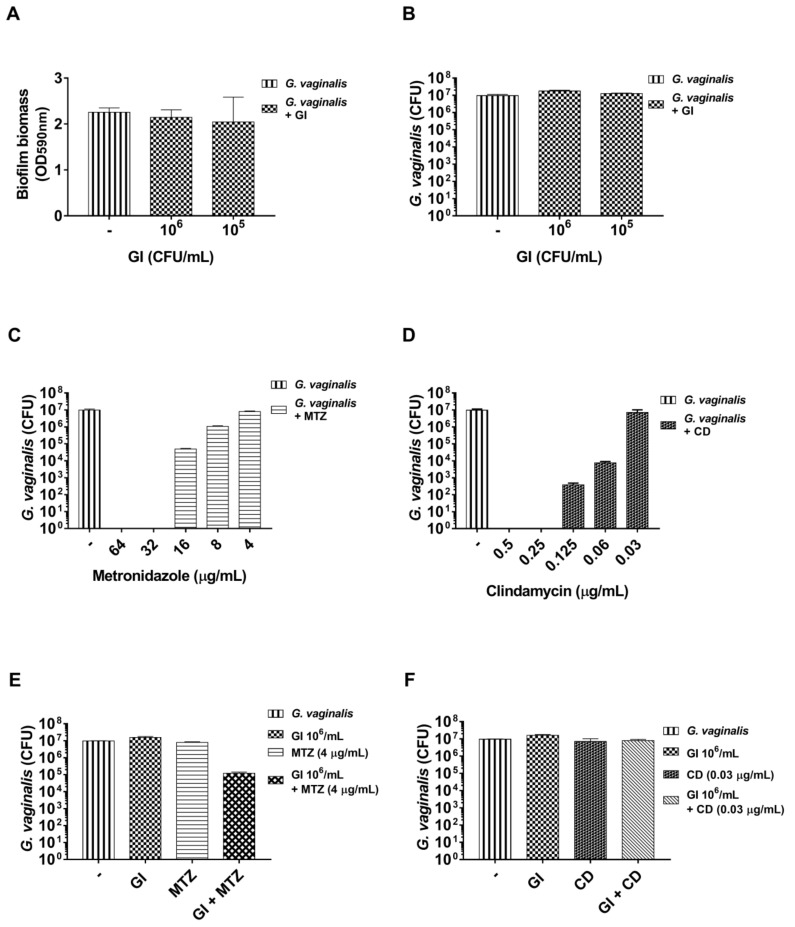
Effect of *S. cerevisiae* CNCM I-3856 (GI)-antibiotic combination on preformed *G. vaginalis* biofilm: (**A**) *G. vaginalis* (10^8^ CFU/mL) was incubated for 24 h to develop mature biofilm. After incubation, the medium was removed, GI was added at different concentrations, and the co-cultures were incubated for a further 24 h as described in the Materials and Methods section. Biofilm formation has been evaluated by crystal violet staining (OD, optical density). Data are the mean ± SEM from three experiments, each with n = 3. (**B**) *G. vaginalis* biofilm viability was determined by a colony forming units (CFU) assay. Data are the mean ± SEM from one experiment with n = 3. (**C**,**D**) *G. vaginalis* (10^8^ CFU/mL) was incubated for 24 h to develop mature biofilm. After incubation, the medium was removed and metronidazole (MTZ) (**C**) or clindamycin (CD) (**D**) were added to the preformed biofilm and incubated for another 24 h to determine the minimal biofilm bactericidal concentration as described in the Materials and Methods section. Data are the mean ± SEM from one experiment with n = 3. (**E**) *G. vaginalis* (10^8^ CFU/mL) preformed biofilm was incubated with GI (10^6^ CFU/mL), metronidazole (4 µg/mL), or metronidazole (4 µg/mL) plus GI (10^6^ CFU/mL) in the same experimental conditions as above described, and *G. vaginalis* biofilm viability was determined by a CFU assay. Data are the mean ± SEM from one experiment with n = 3. (**F**) *G. vaginalis* (10^8^ CFU/mL) preformed biofilm was incubated with GI (10^6^ CFU/mL), clindamycin (0.0312 µg/mL), or clindamycin (0.0312 µg/mL) plus GI (10^6^ CFU/mL) in the same experimental conditions as above described. *G. vaginalis* biofilm viability has been determined by a colony forming unit (CFU) assay. Data are the mean ± SEM from one experiment with n = 3.

In order to evaluate if GI can potentiate the antibiotic effect on preformed *G. vaginalis* biofilms, we first evaluated the suboptimal doses of the probiotic on biofilm biomass and viability. Our results show that GI at doses of 10^6^ and 10^5^ CFU/mL did not produce reduction both of total biofilm biomass and of *G. vaginalis* biofilm viability (Figure 5A,B).

Then, the MIC values for metronidazole and for clindamycin were evaluated. In our experimental conditions, metronidazole exhibited an MIC of 16 µg/mL and clindamycin exhibited an MIC of 0.0625 µg/mL. The minimal biofilm bactericidal concentration (BBC) for both antibiotics was then determined. To this end, 24 h preformed *G. vaginalis* biofilm was incubated for another 24 h with two-fold serial dilutions of metronidazole or clindamycin and *G. vaginalis* biofilm viability was determined by a CFU assay. As reported in Figure 5C,D, metronidazole and clindamycin exhibited BBC values of 32 µg/mL and 0.25 µg/mL, respectively, and the first dose that did not significantly reduce *G. vaginalis* viability was 4 µg/mL for metronidazole and 0.0312 µg/mL for clindamycin. These suboptimal concentrations have been selected for subsequent experiments.

The results reported in Figure 5E show that GI (10^6^ CFU/mL) was able to potentiate the metronidazole activity and produced a very strong reduction of biofilm viability. Conversely, the results obtained from the association of GI with clindamycin showed that the combination of suboptimal doses of GI (10^6^ CFU/mL) with suboptimal doses of clindamycin (0.0312 µg/mL) did not produce effects on *G. vaginalis* biofilm viability (Figure 5F).

## 4. Discussion

BV is a gynecological infection associated with the presence of a highly organized polymicrobial biofilm adhering to vaginal epithelial cells, with *G. vaginalis* having a key role in the constitution of this biofilm [28,66,67,68,69,70]. The establishment of this biofilm is a required step for BV initiation and development [16,71,72]. Conventional antibiotic therapy constitutes a first-line response but is ineffective in a high proportion of women, explaining the high recurrence rate [23,25,73]. Besides being associated with a number of side effects, including nausea, vomiting, and gastrointestinal complaints [23,74,75,76], antibiotic therapy, especially when prolonged or frequently repeated, can result in the development of resistant pathogens [18,23,25] without restoring a healthy vaginal microbiota [77]. Complementary and/or alternative therapeutic approaches for BV are therefore necessary. In the last years, several studies have reported the beneficial effect of potential anti-biofilm agents such as DNase [33]; retrocyclins [31]; antibacterial enzymes, peptides, antiseptics, and tensides [26,35]; quorum-sensing inhibitors subtilosin [32], and benzoyl peroxide [34]. Probiotics also seem to be a valid adjuvant or alternative option to the conventional therapeutic strategies both for prevention and for treatment of several human diseases such as bacterial vaginosis [37,38,43,78,79,80]. To date, there are limited studies on the effect of probiotics in inhibiting *G. vaginalis* biofilm formation or disaggregation [40,42,43] and none have been reported for *S. cerevisiae*. In this study, *S. cerevisiae* CNCM I-3856 and lactic acid bacteria *L. rhamnosus* GG (ATCC 53103) probiotics were tested for their ability to inhibit biofilm formation and to disaggregate mature *G. vaginalis* biofilms.

Recently, our research group demonstrated the beneficial effect of probiotics on vaginal candidiasis [46,47] and on BV [49,80]. In particular, in an experimental murine model of BV, we showed that *S. cerevisiae* CNCM I-3856 was able (i) to inhibit *G. vaginalis* adhesion to vaginal epithelium, (ii) to displace *G. vaginalis* adhered to epithelial cells, (iii) to reduce *G. vaginalis* vaginal load and sialidase activity, and iv) to decrease epithelial exfoliation [49].

**Figure 6:**
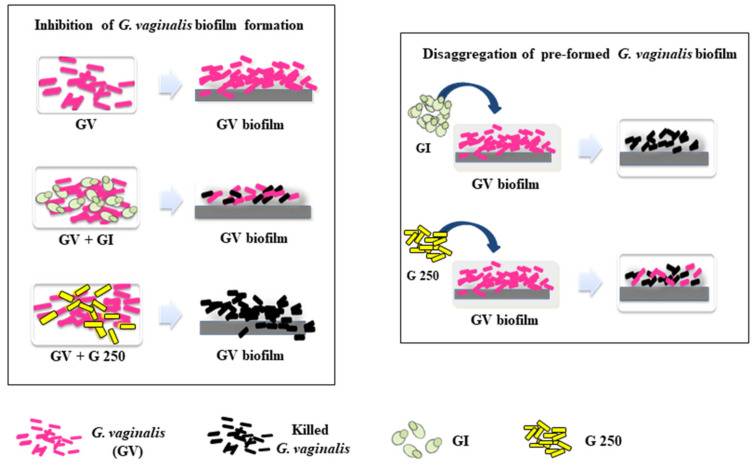
Representation of anti-*G. vaginalis* biofilm activity of *S. cerevisiae* CNCM I-3856 (GI) and *L. rhamnosus* GG (G250).

Here, for the first time, we demonstrated that *S. cerevisiae* CNCM I-3856 as well as *L. rhamnosus* GG were able i) to significantly inhibit *G. vaginalis* biofilm formation, ii) to markedly reduce *G. vaginalis* viability among the biomass constituting the biofilm, iii) to induce disaggregation of preformed biofilms, and iv) to kill a consistent amount of bacterial cells in a *G. vaginalis* preformed biofilm (Figure 6).

Furthermore, *S. cerevisiae* strongly potentiates the antibiotic effects on *G. vaginalis* biofilm viability.

Several mechanisms [81] may interfere with biofilm formation and maintenance such as competition for nutrients and for binding sites, contact-dependent growth inhibition, secretion of inhibitory molecules, and inhibition of cell-to-cell communication. Given that we recently demonstrated that *S. cerevisiae* CNCM I-3856 (GI) is unable to co-aggregate with *G. vaginalis* [49], it is possible that *S. cerevisiae* CNCM I-3856 inhibition of *G. vaginalis* biofilm formation can be ascribed to biological instead of mechanical effects. Indeed, previous studies demonstrated that members of the *Saccharomyces* genus can produce extracellular protease and other compounds able to inhibit the growth of many pathogenic bacterial species [82,83]. In addition, given that the presence of the sialidase gene is associated with *G. vaginalis* biofilm formation [84], the capacity of *S. cerevisiae* CNCM I-3856 in inhibiting sialidase activity [49] could account for the observed effect.

Co-aggregation of pathogenic microorganisms is one of the mechanisms used by lactobacilli to maintain a healthy vaginal environment [65], and it is recognized as the one by which lactobacilli can exert their probiotic effects [65,85,86,87]. Indeed, differently from *S. cerevisiae* CNCM I-3856, *L. rhamnosus* GG was able to co-aggregate with *G. vaginalis*, suggesting that, by this interaction, *L. rhamnosus* GG produces around the *G. vaginalis* a microenvironment with an high concentration of antimicrobial substances [88] that not only may inhibit *G. vaginalis* growth but also may cause its death.

In the current study, we also demonstrated that *S. cerevisiae* CNCM I-3856 (10^8^ and 10^7^ CFU/mL) produced a significant disaggregation of the preformed *G. vaginalis* biofilm. This is consistent with our recent work showing that *S. cerevisiae* CNCM I-3856 is effective in displacing *G. vaginalis* adhered to vaginal and cervix epithelial cells [49]. Of note, *S. cerevisiae* CNCM I-3856 killed almost 100% of *G. vaginalis* cells in the biomass. This effect against *G. vaginalis* could be accounted for by two putative mechanisms. First, *S. cerevisiae* CNCM I-3856 can degrade the extracellular matrix by secreting enzymes [82] such as amylase, protease, and lipase that favor penetration of the yeast into the biofilm, allowing it to displace *G. vaginalis* from the abiotic surface. Second, the probiotic can have an antimicrobial activity by secreting specific compounds [82,83].

*L. rhamnosus* GG was also able to significantly disaggregate the *G. vaginalis* biofilm but only at 10^7^ CFU/mL, probably because low doses facilitate penetration into the preformed biofilm. On the other hand, it was able to significantly reduce *G. vaginalis* viability in the biofilm in a dose-dependent manner. This is likely due to the production of antimicrobial compounds able to kill *G. vaginalis* cells [89,90,91].

In this study, we observed that the same doses of *L. rhamnosus GG* (10^7^ CFU/mL) induced different effects in biofilm formation and biofilm disaggregation. This could be related to different mechanisms involved. Indeed, it is possible that low doses of *L. rhamnosus* GG are not sufficient to induce *G. vaginalis* coaggregation but could favor its penetration inside the biofilm, thus facilitating disruption.

Biofilms play an important role in conferring to microorganisms a marked increase in tolerance to antimicrobial agents [21]. The development of therapeutic combinations of antibiotics and probiotics may represent a promising alternative strategy to compromise biofilm maintenance, in particular, the use of yeast-based probiotics since yeast cannot promote the spread of antimicrobial resistance because exchange of antibiotic resistance genes with bacteria is unlikely. In our experimental system, the coadministration of *S. cerevisiae* CNCM I-3856 with metronidazole improved the efficacy of an otherwise suboptimal antibiotic dose. These results are encouraging as the use of low dosages of antibiotics could decrease side effects and the development of antibiotic resistant biofilms.

Our study presents, however, some limitations due to the fact that the biofilm obtained in this in vitro model is only constituted by *G. vaginalis* whereas the biofilm of bacterial vaginosis is a polymicrobial biofilm made up of *G. vaginalis* and other anaerobic bacteria, such as *Prevotella bivia* and *Atopobium vaginae*, which synergistically contribute to its formation [72,92].

Here, we demonstrate that both *S. cerevisiae* CNCM I-3856 and *L. rhamnosus* GG probiotics are able to inhibit *G. vaginalis* biofilm formation and to induce disaggregation of preformed biofilms. More importantly, we demonstrate that these probiotics can kill *G. vaginalis* during biofilm formation and can destroy almost completely *G. vaginalis* cells into mature biofilm. In addition, *S. cerevisiae* CNCM I-3856 strongly potentiate antibiotic efficacy. This should be considered important findings considering the difficulty to eradicate preformed biofilms from different bacteria [27,93,94,95].

## 5. Conclusions

The treatment of biofilm-involving infections is currently known to be very complicated partly due to the inefficiency of conventional antibiotic treatments in overcoming such infections.

Here, we proposed a novel and alternative approach for inhibition of *G. vaginalis* biofilm development and for disaggregation of the preformed one. Both probiotics evaluated were shown to be able to inhibit and to disrupt the *G. vaginalis* biofilm.

Furthermore, our results suggest that *S. cerevisiae* CNCM I-3856 is a good potential candidate to be used in combination with metronidazole for the treatment of bacterial vaginosis.

## Figures and Tables

**Table 1:** Scores of *G. vaginalis* and *L. rhamnosus* GG (G 250) co-aggregation.

Co-Aggregation Scores *			
	*G. vaginalis*	G 250	*G. vaginalis* + G 250
EXP 1	0	0	3
EXP 2	0	1	3
EXP 3	0	2	3.5
**Mean score**	**0**	**1**	**3.16**

* Scores from 0 (no aggregation) to 4 (maximum aggregation).
